# Differences in survival, virulence and biofilm formation between sialidase-deficient and W83 wild-type *Porphyromonas gingivalis* strains under stressful environmental conditions

**DOI:** 10.1186/s12866-017-1087-2

**Published:** 2017-08-18

**Authors:** Xiaoyu Xu, Tong Tong, Xue Yang, Yaping Pan, Li Lin, Chen Li

**Affiliations:** 1 Department of Periodontics, School of Stomatology, China Medical University, No.117 Nanjing North Street, Heping District, Shenyang, Liaoning China; 2 Department of Stomatology, Anshan Shuangshan Hospital, Anshan, Liaoning China; 3 Shenyang Medical College, Shenyang, Liaoning China

## Abstract

**Background:**

*Porphyromonas gingivalis* is a major causative pathogen of chronic periodontitis. Within the inflammatory microenvironment, there exists extreme pH values, elevated temperatures and oxidative stress. Pathogens adapt to these stressful environmental conditions by regulating the transcription of virulence genes, modifying themselves with macromolecules and by aggregating and entering into a biofilm growth phase. Our previous study showed that the *P. gingivalis* sialidase can help cells obtain sialic acid from the environment, which is used to modify macromolecules on the surface of *P. gingivalis* cells. In this study, we compared the survival, virulence factors and biofilm formation of a sialidase-deficient strain (ΔPG0352) and the wild-type *P. gingivalis* W83 strain under various pH values, temperatures and oxidative stress conditions to identify the roles of sialidase in the adaptation of *P. gingivalis* to stressful conditions.

**Results:**

Compared to the growth of the *P. gingivalis* W83 strain, the growth of the △PG0352 was more inhibited by oxidative stress (0.25 and 0.5 mM H_2_O_2_) and exhibited greater cell structure damage when treated with H_2_O_2_ as assessed by transmission electron microscopy. Both Lys-gingipain (Kgp) and Arg-gingipain (Rgp) activities were lower in the ΔPG0352 than those in the *P. gingivalis* W83 strain under all the assayed culture conditions. The lipopolysaccharide (LPS) activity of the W83 strain was higher than that of the ΔPG0352 under acidic conditions (pH 5.0), but no differences between the strains were observed under other conditions. Compared to the biofilms formed by *P. gingivalis* W83, those formed by the ΔPG0352 were decreased and discontinuous under acidic, alkaline and oxidative stress conditions.

**Conclusion:**

Compared to the *P. gingivalis* W83 strain, the survival, virulence and biofilm formation of the ΔPG0352 were decreased under stressful environmental conditions.

## Background

Periodontal diseases are complex, multifactorial and polymicrobial inflammatory diseases. They are characterized by the destruction of the supporting tissues around the teeth. The primary pathological changes associated with periodontal diseases, especially chronic periodontitis, are periodontal inflammation, loss of periodontal epithelial attachment, periodontal pocket formation and alveolar bone resorption, which ultimately lead to loosening and exfoliation of the teeth. Data collected by the World Health Organization (WHO) indicate that periodontal diseases affect 10-15% of adults worldwide [1]. It is noteworthy that periodontal diseases have a bidirectional relationship with systemic diseases, such as cardiovascular disease [2], diabetes [3], and rheumatoid arthritis [4], severely affecting the quality of human life.

During the progression of periodontal disease, subgingival microorganisms survive in the inflammatory microenvironment, protecting themselves from the deleterious effects of extreme pH values, elevated temperatures and oxidative stress. Microorganisms must overcome these harsh conditions to colonize or invade the host and can cause inflammation. To survive under these stressful microenvironmental conditions, bacteria will undergo cellular and physiological changes that include regulating the transcription of virulence genes, modifying themselves with macromolecules through reactions such as sialylation and glycosylation and by aggregating and entering into a biofilm growth phase.

*Porphyromonas gingivalis* is a black-pigmented, gram-negative anaerobe that is the major subgingival etiologic agent contributing to chronic periodontitis [5]. Its pathogenicity is attributed to an array of potential virulence factors, such as cysteine proteinases (gingipains), lipopolysaccharide (LPS), and fimbriae that allow *P. gingivalis* to attach to host tissues and resist the host innate immune system, ultimately leading to periodontal tissue destruction [6]. Recently, the role of sialidases in the pathogenesis of pathogens has attracted increased attention. Sialidases (neuraminidase) are glycosyl hydrolases that can cleave the connection between glycosylated substrates and sialic acid via a hydrolysis reaction. In a previous study, we confirmed that *PG0352* is the sole sialidase-encoding gene in *P. gingivalis* W83, and we constructed a sialidase-deficient mutant strain (∆PG0352) by homologous recombination. We found that the deletion of *PG0352* influenced biofilm formation and capsule biosynthesis and decreased the pathogenicity of *P. gingivalis* in a mouse subcutaneous abscess model [7].

The survival of *P. gingivalis* in the inflammatory microenvironment requires that it survive under various stresses, including different pH values, elevated temperatures, and oxidative stress. Some *P. gingivalis* mutations were observed to influence its survival, especially under these stress conditions. An investigation by Yuan et al. showed that a *clpB* (encoding a component of the stress response) mutant strain of *P. gingivalis* W83 demonstrated an increased sensitivity to heat stress, but not to hydrogen peroxide and extreme pH values [8]. McKenzie et al. indicated that the inactivation of *pg1372* (encoding a protein with DNA-binding properties) virtually eliminated the ability of *P. gingivalis* to adapt to oxidative stress [9]. Dou et al. showed that a *PG2212* (encoding a zinc finger protein) isogenic mutant strain of *P. gingivalis* exhibited an increased sensitivity to H_2_O_2_ [10]. In this study, we cultured *P. gingivalis* W83 and its sialidase-deficient mutant strain under stressful environmental conditions, including various pH values, temperatures and oxidative stress conditions, and compared the growth, morphology, tolerance, virulence factors and biofilm formation of these two strains to investigate the roles of sialidase in the adaptation of *P. gingivalis* to these stressful conditions.

## Methods

### Bacterial strains and culture conditions

*P. gingivalis* W83 and △PG0352 were used in this study. The *P. gingivalis* W83 strain was obtained from the Department of Oral Biology at China Medical University and the △PG0352 was constructed in our previous study [7]. Both strains were cultured anaerobically (10% CO_2_, 10% H_2_, and 80% N_2_) in trypticase soy broth (TSB, BD Diagnostic Systems, Aparks, MD) supplemented with 5 μg/mL hemin, 1 μg/mL menadione and 1 mg/mL yeast extract. For blood agar plates, TSB medium was supplemented with 1.5% agar and 5% sheep blood(Beiruite Bio-technology Co.,Ltd., Beijing, China).

### Stress experiments

For all stress-related experiments, the *P. gingivalis* W83 and △PG0352 strains were incubated until they reached exponential phase (OD_600_ = 0.8-1.0).

1. pH-induced stress. The pH of the TSB medium was adjusted to 5.0, 7.2 or 9.0 by the addition of 1 M NaOH or 0.5 M HCl. Bacterial cells were centrifuged at 3000×g for 8 min at 4 °C, then the pellets were washed with phosphate-buffered saline (PBS) and resuspended in TSB medium at different pH values [8].
2. Temperature-induced stress. First, *P. gingivalis* W83 and △PG0352 cultures were treated as described above, then cells were resuspended in freshly supplemented TSB. Resuspensions of each strain were divided into three portions that were then cultured at 41, 37 or 34 °C [11].
3. Hydrogen peroxide-induced oxidative stress. First, *P. gingivalis* W83 and △PG0352 cultures were treated as described above. Next, hydrogen peroxide (Sigma-Aldrich, St. Louis, MO, USA) was added to the TSB medium and both strains were incubated at final concentrations of 0.1, 0.25, 0.5 or 1 mM H_2_O_2_. Final concentrations of hydrogen peroxide (Sigma-Aldrich, St. Louis, MO, USA) were obtained by diluting a 3% solution (8.8 mM) to the appropriate molarity in TSB medium [9]. Cell cultures that were not treated with hydrogen peroxide were used as controls.

### Growth curves

The *P. gingivalis* W83 and ΔPG0352 strains were incubated under different culture conditions for 24 h. The growth curves of both strains under pH- and temperature-induced stress were generated by recording the absorbance at 600 nm (OD_600_) after 0, 4, 8, 12 and 24 h of culturing using a spectrophotometer (Bio-Rad, USA). The growth curves of the strains grown under oxidative stress were generated by measuring the OD_600_ after 0, 1, 4, 8 and 12 h of culturing.

### Transmission electron microscopy

*P. gingivalis* W83 and ΔPG0352 cultures were centrifuged and washed three times with PBS. The pellets were fixed with 2.5% glutaraldehyde at 4 °C overnight [12]. Next, cells were washed three times with PBS and suspended in an osmium tetroxide solution for 2 h. The washed cell pellets were then gradually dehydrated in a series of ethanol (30%, 50% and 70%) and acetone (80%, 90% and 100%) solutions for 30 min each. Finally, the pellets were embedded in Epox 812 resin. Thin sections were cut and double-stained with uranyl acetate-lead citrate then were viewed under a transmission electron microscope (TEM) (H7650 Hitachi, Japan) at 70,000× magnification.

### Gingipain activity assay

Nα-Benzoyl-DL-arginine p-nitroanilide hydrochloride (BAPNA) and N-(p-Tosyl)-Gly-Pro-Lys 4-nitroanilide acetate salt (both purchased from Sigma-Aldrich, St. Louis, MO, USA) were used as substrates to determine the activities of Arg-gingipain (Rgp) and Lys-gingipain (Kgp), respectively, according to a described previously method [13]. In brief, the *P. gingivalis* W83 and ΔPG0352 strains were cultured (16 h for pH-induced and temperature-induced stresses and 8 h for hydrogen peroxide-induced oxidative stress); then, cells were harvested by centrifugation at 5000×g for 5 min at 4 °C. Next, the pellets were suspended in 2 mL of reaction buffer, then 100 μL of the prepared pellet suspensions were aliquoted into an ice-cold 96-well microtiter plate. After a 10 min incubation at 37 °C, 100 μL of a 0.5 mM substrate solution was added and the OD_405_ was measured.

### Preparation and activity measurement *P. gingivalis* LPS

LPS was isolated from 50 mL of bacterial cultures using the TRI-Reagent protocol as previously described [14]. *P. gingivalis* strains were cultured under varying conditions to mid-log phase, centrifuged at 6500 rpm for 20 min and the pellets were resuspended in TRI-reagent (Takara Bio Inc., Japan). Next, a 1/5 volume chloroform was added and mixed, the mixtures were centrifuged at 12,000 rpm for 10 min, and the top aqueous layers were retained as “crude LPS” extracts. The crude LPS extracts were washed with 1 mL of cold 0.35 M MgCl_2_ in 95% ethanol and centrifuged at 5000 rpm for 5 min at 4 °C. The pellets were washed twice more with 1 mL of cold 95% ethanol and once with 1 mL of cold 100% ethanol; then, the pellets were air-dried. To remove contaminating phospholipids, LPS extracts of each strain were resuspended to a 1% (*w*/*v*) solution in a 2:1 chloroform-methanol solution, centrifuged at 5000 rpm for 5 min at 4 °C, and air-dried. The final products were resuspended in water to attain the same concentrations among the different samples. LPS activity was measured using a Chromogenic End-point Tachypleus Amebocyte Lysate Kit (Chinese Horseshoe Crab Reagent Manufactory, China) according to the manufacturer’s instructions. Briefly, the tachypleus amebocyte lysate solutions, chromogenic substrates and azo reagents were successively added to diluted standards and LPS solutions and incubated for the appropriate time at 37 °C. Next, the mixtures were transferred to a 96-well immunoassay plate. Dilutions of all standards and samples were performed in triplicate. Lastly, a microplate reader (Infinite M200, TACON, Germany) was used to immediately measure the optical densities of the samples at 545 nm. Standard curves were generated by plotting the mean optical density and the activity of each standard dilution and each LPS solution.

### Biofilm formation observed by confocal microscopy

Biofilms were grown and microscopically examined in 35 × 10 mm^2^ polystyrene cell culture plates (Corning, Netherlands) that were coated with artificial saliva (Guangzhou Kodak Adhesives Co. Ltd., China). Cell suspensions (2 mL; 5 × 10^6^ CFU/mL) were aliquoted into polystyrene plates and cultured under varying conditions as described above for 4 days. The plates were washed twice with sterile PBS, and the biofilms were stained with a bacterial viability kit (Live/Dead BacLight bacterial viability kit L-7007; Molecular Probes, US) for 15 min in the dark then were washed twice more with sterile PBS. The polypropylene wells were detached from each other and the slides were air-dried. The biofilms were imaged with a confocal laser scanning microscope (CLSM) (Olympus FV1000, Japan) at an excitation wavelength of 488 nm. The three-dimensional structure of the biofilm was reconstructed from CLSM images using the built-in software (Olympus FV1000 Viewer, Japan). The thickness and density of each biofilm was observed by serially scanning layers from the inside (plaque biofilm and slide surface) to the outside (plaque biofilm-free surface) at a specific interval of 1.5 μm and measurements were subsequently made using ImageJ.

### Statistical analysis

All experiments were performed in triplicate for each strain under each condition and were repeated at least three times. The data were analyzed by ANOVA to compare the differences of one strain grown under various conditions. Independent-sample t-tests were used to analyze the differences between the two strains grown in the same culture conditions. The level of significance was set at 0.05.

## Results

### Compared to *P. gingivalis* W83, the ΔPG0352 strain exhibited reduced survival under oxidative stress

**Fig. 1:**
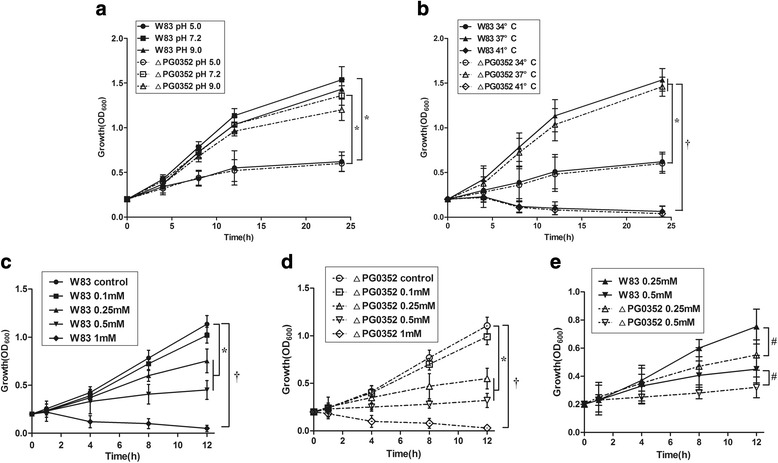
The growth curves of the *P. gingivalis* W83 and ΔPG0352 strains grown under varying pH values (**a**), temperatures (**b**) and oxidative stress conditions (**c-e**). ^*^ *P* < 0.05;^†^ *P* < 0.01

Both the *P. gingivalis* W83 and ΔPG0352 strains grew significantly more slowly under acidic conditions (pH 5.0) than in the normal medium (pH 7.2) (*P* < 0.05), but alkaline conditions (pH 9.0) did not affect the growth of either strain. There were no differences between the growth rates of the *P. gingivalis* W83 and ΔPG0352 strains under pH-induced stress. (Fig. 1a). For the growth curves at different temperatures, the growth rates of both strains were considerably decreased at 34 °C compared to 37 °C, while both strains barely grew at 41 °C. At a given temperature, the growth rate of the *P. gingivalis* W83 strain was not significantly different from that of the ΔPG0352 (Fig. 1b). For the growth curves under oxidative stress, a concentration of 0.1 mM H_2_O_2_ had no effect on the growth of either the *P. gingivalis* W83 or ΔPG0352 strains. Final concentrations of 0.25 mM and 0.5 mM H_2_O_2_ resulted in a significant decrease in the growth rates of both strains, while both strains were unable to grow after being treated with 1 mM H_2_O_2_ (Fig. 1c and d). Compared to the *P. gingivalis* W83 strain, the ΔPG0352 grew more slowly at final concentrations of 0.25 and 0.5 mM H_2_O_2_ (*P* < 0.05), and there were no significant differences between the *﻿﻿t﻿wo﻿﻿ ﻿P. gingivalis* strains at final concentrations of 0.1 and 1 mM H_2_O_2_ (Fig. 1e).

### Compared to *P. gingivalis* W83 strain, the cell membrane of the sialidase-deficient mutant was more easily damaged by oxidative stress

**Fig. 2:**
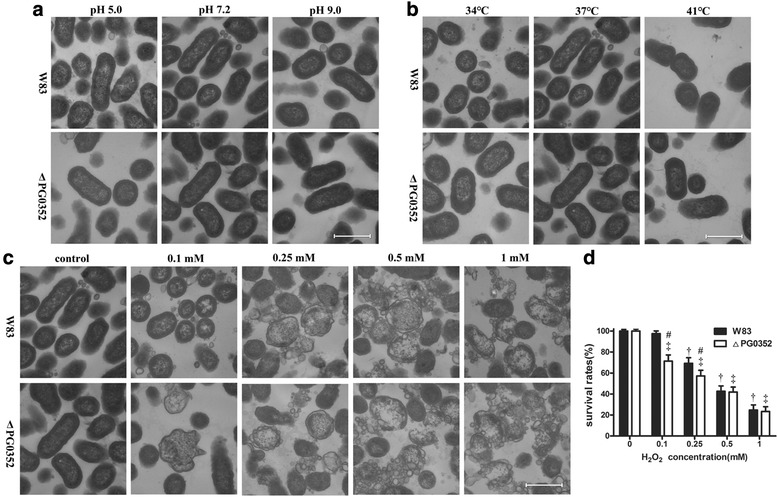
TEM images of *P. gingivalis* the W83 and ΔPG0352 strains cultured under varying pH values (**a**), temperatures (**b**) and oxidative stress conditions (**c**). Magnification: 70,000×; bar: 500 nm. (**d**) Survival rates of both strains cultured under oxidative stress; ^†^ versus the *P. gingivalis* W83 strain grown under normal culture conditions, *P* < 0.05; ^‡^ versus the ΔPG0352 mutant grown under normal culture conditions, *P* < 0.05; ^#^ versus the *P. gingivalis* W83 strain under the same culture conditions, *P* < 0.05

Under normal growth conditions, the cell membranes of both the *P. gingivalis* W83 and ΔPG0352 strains were intact and clearly observable by TEM. The cell structures of both strains showed no significant differences at different pH values (Fig. 2a) or temperatures compared to cells grown under normal culture conditions (Fig. 2b). At a concentration of 0.1 mM H_2_O_2_, the cell structure of the *P. gingivalis* W83 strain was not damaged, while nuclear separation occurred in ΔPG0352 cells. Final concentrations of 0.25, 0.5 and 1 mM H_2_O_2_ caused a discontinuity of the cellular membrane, cytoplasm shrinking and plasmolysis in both *P. gingivalis* W83 and ΔPG0352 cells. As the concentration of H_2_O_2_ increased, the cell structures were damaged with increasing severity. For both the *P. gingivalis* W83 and ΔPG0352 strains, cytoplasm vacuoles were degenerated and intact cells were rarely observed at a final concentration of 1 mM H_2_O_2_ (Fig. 2c). The survival rates of the ΔPG0352 mutant in 0.1 mM and 0.25 mM H_2_O_2_ were significantly lower than those of *P. gingivalis* W83. At concentrations of 0.5 mM and 1 mM H_2_O_2_, the survival rates of both strains decreased, but there were no significant differences between both *P. gingivalis* W83 and ΔPG0352 strains under same conditions. (Fig. 2d).

### Gingipains and LPS activities in *P. gingivalis* W83 and ΔPG0352 strains varied under certain stressful culture conditions

**Fig. 3:**
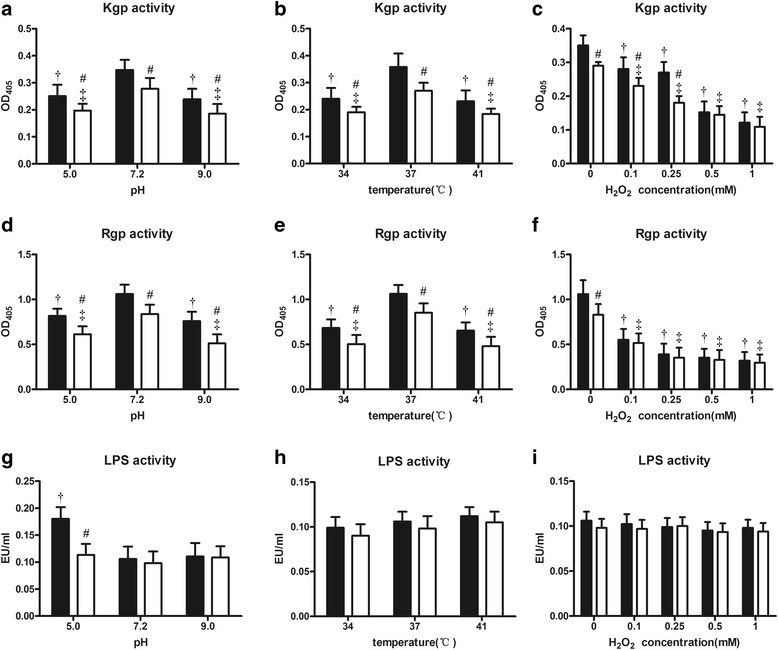
Comparison of virulence factors between the *P. gingivalis* W83 (■) and ΔPG0352 (□) strains cultured under varying conditions. ^†^ the difference in the *P. gingivalis* W83 strain is significant between normal and stressful conditions, *P* < 0.05; ^‡^ the difference in the ΔPG0352 mutant is significant between normal and stressful conditions, *P* < 0.05; ^#^ versus *P. gingivalis* W83 under the same culture conditions, *P* < 0.05

Under all stress conditions assayed, including acidic or alkaline conditions (pH 5.0 or 9.0), extreme temperatures and oxidative stress, the activities of Kgp and Rgp in the *P. gingivalis* W83 and ΔPG0352 strains were lower than those in normal growth conditions. The Kgp activities for the ΔPG0352 were lower than those for the *P. gingivalis* W83 strain under all stressful conditions except with 0.5 mM and 1 mM H_2_O_2_ (Fig. 3a-c). The Rgp activities of the ΔPG0352 were lower than those of the *P. gingivalis* W83 strain under acidic or alkaline conditions and extreme temperatures, but no significant difference in Rgp activity was observed between these strains under any oxidative stress condition tested (Fig. 3d-f). The LPS activities of both the *P. gingivalis* W83 and ΔPG0352 strains did not change, and there were no differences between the strains under stressful conditions except for acidic conditions. Under acidic conditions (pH 5.0), the LPS activity of *P. gingivalis* W83 was significantly higher than that of the ΔPG0352 and was significantly higher than that of the *P. gingivalis* W83 strain grown under any other culture condition (Fig. 3g-i).

### Compared to *P. gingivalis* W83 strain, the biofilm formation of the ΔPG0352 was severely decreased under stressful conditions

**Fig. 4:**
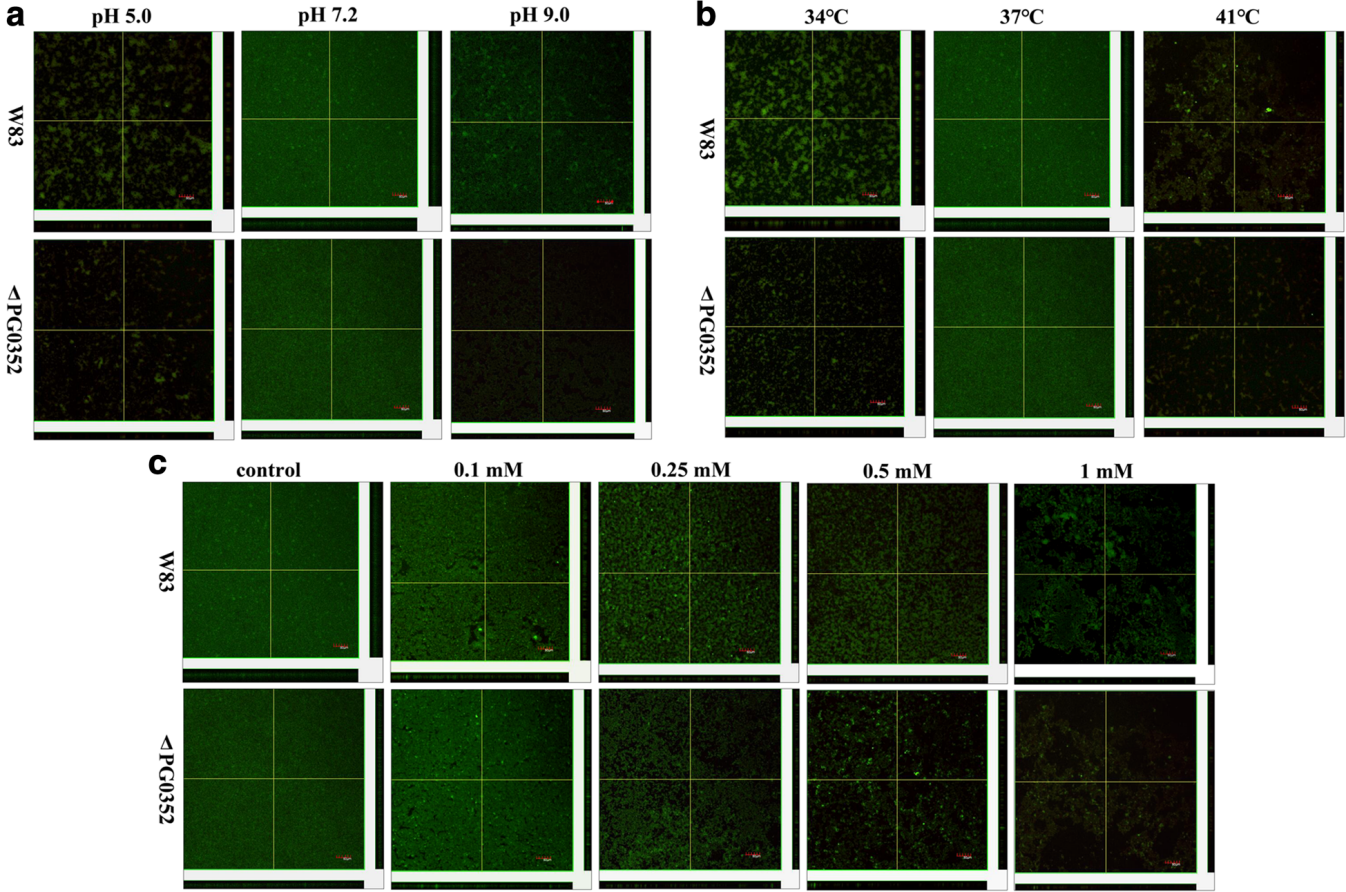
Confocal microscopy images of the *P. gingivalis* W83 and ΔPG0352 biofilms (stained with live/dead bacterial viability kit) cultured under varying pH values (**a**), temperatures (**b**) and oxidative stress conditions (**c**). Bar: 50 μm

**Fig. 5:**
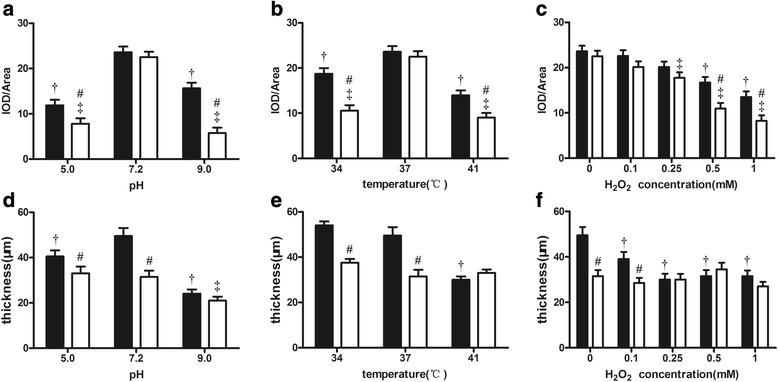
Comparison of the mean thickness (**a, b** and **c**) and fluorescence density (**d, e** and **f**) of biofilms between the *P. gingivalis* W83 (■) and ΔPG0352 (□) strains cultured under stressful conditions. † for *P. gingivalis* W83, the difference is significant between normal and stressful conditions, *P* < 0.05;. ^‡^ for the ΔPG0352 mutant, the difference is significant between normal and stressful conditions, *P* < 0.05; ^#^ versus *P. gingivalis* W83 under the same culture conditions, *P* < 0.05

Under normal conditions, the biofilms formed by the *P. gingivalis* W83 and ∆PG0352 strains showed a uniform and dense fluorescence signal, although the biofilm by the ∆PG0352 was thinner than that of the *P. gingivalis* W83 strain. Biofilm formation by both the *P. gingivalis* W83 and ∆PG0352 strains decreased under acidic conditions (pH 5.0) and decreased more pronounced under alkaline conditions (pH 9.0). Biofilms of both strains were scattered in bacterial clumps, with the ∆PG0352 mutant forming less biofilm than that of the *P. gingivalis* W83 strain under both acid and alkaline conditions (Figs. 4a, 5a and d). An increase or decrease in temperature resulted in decreased biofilm formation for both strains, and less biofilm was formed by the ∆PG0352 mutant than by the *P. gingivalis* W83 strain (Figs. 4b, 5b and e). At a hydrogen peroxide concentration of 0.1 mM, both *P. gingivalis* strains exhibited continuous biofilm formation, but they were thinner than those generated under normal conditions. As the hydrogen peroxide concentration increased, gaps and pore structures became increasingly prevalent and finally became bacterial mass in the biofilms of both the *P. gingivalis* W83 and ∆PG0352 strains. Dense biofilm fluorescence signals for the ∆PG0352 were less common than those for the *P. gingivalis* W83 strain at final concentrations of 0.5 and 1 mM H_2_O_2_ (Figs. 4c, 5c and f).

## Discussion

In this study, the growth curves of the *P. gingivalis* W83 and ∆PG0352 strains grown in normal and stressful conditions revealed that both strains grew slower at lower temperatures and under oxidative stress; compared to ∆PG0352, the wild-type strain was more sensitive to hydrogen peroxide but not to extreme pH values and temperatures. Bacteria primarily grew slower under sub-optimal temperatures because the activities of enzymes associated with their growth decrease and the enzymatic reactions slow down [15], while *P. gingivalis* grew slower under oxidative stress because of protein degeneration and cell death. Compared to *P. gingivalis* W83, the ∆PG0352 was more sensitive to hydrogen peroxide. *P. gingivalis* is an anaerobic microorganism and enzymes that detoxify oxygen are important in allowing it to inhabit oxygenated environments. Diaz PI et al. found that alkyl hydroperoxide reductase contributed to the tolerance of *P. gingivalis* to moderate levels of H_2_O_2_ [16]. In addition, Lijima R et al. found that sialic acid played a role as a reactive oxygen scavenger, protecting cells against hydrogen peroxide [17]. *P. gingivalis* cannot produce sialic acid but uses sialidase to cleave sialoglycoconjugates in the environment to produce free sialic acid [18, 19]. Thus, the sialidase-deficient mutant strain cannot release sialic acid to detoxify hydrogen peroxide and is therefore more sensitive to hydrogen peroxide than the W83 strain. Another reason for the oxidative stress sensitivity of the sialidase-deficient mutant is that it cannot synthesize an intact capsule; thus, the cell membrane of the sialidase-deficient mutant is easily damaged under oxidative stress.

Maintaining cell structure is essential for *P. gingivalis* survival, therefore we performed TEM to assess differences in structure between the *P. gingivalis* W83 and ∆PG0352 strains that were grown in varying stressful culture conditions. At different pH values or varying temperatures there was no significant difference in the cell structures of either strain compared to those grown under normal culture conditions. As the concentration of H_2_O_2_ increased, the balance of osmotic pressure between the inner and outer membranes was gradually lost, damaging the cell structure. The cell structure of the ∆PG0352 mutant was more easily damaged than that of the *P. gingivalis* W83 strain under oxidative stress. There are two possible reasons for this observation. The first reason is that sialidase deficiency in *P. gingivalis* influences capsule biosynthesis. Sialidase can cleave sialoglycoconjugates in the environment and produce free sialic acid, which are used for capsule biosynthesis [20]. The capsule contributes to the maintenance of the cell integrity. The ∆PG0352 mutant would produce a defective capsule due to a lack of sialic acid and would have difficulty maintaining cell structure under stressful conditions. The second reason is that the sialylation of macromolecules on the cell surface may play a role in bacteria maintaining their cell structures [21]. In the ∆PG0352, the macromolecules on the cell surface cannot be sialylated because of the sialidase deficiency. Thus, we speculate that the loss of sialidase may influence the sialylation of membrane molecules, limiting cellular resistance to oxidative stress. Taken together, because of the sialidase deficiency, the biosynthesis of capsule and the sialylation of macromolecule on the cell surface are impacted such that the cell structure of the ∆PG0352 is more easily damaged than that of the *P. gingivalis* W83 strain under oxidative stress.

*P. gingivalis* produces multiple proteases, with the primary ones being a set of cysteine proteases called gingipains, including an arginine-specific protease (Rgp) and a lysine-specific protease (Kgp). Rgp is encoded by two genes, *rgpA* and *rgpB*, and Kgp, which is encoded by a single gene, *kgp* [22]. The gingipains have multiple functions. They disrupt cell-cell and cell-matrix adhesion, induce apoptosis in several cell types [23–25] and contribute to bacterial survival [26]. Our study found that the gingipain activity of the ∆PG0352 was lower than that of the *P. gingivalis* W83 strain under either normal or stressful conditions. Vanterpool et al. showed that a complex of proteins could be involved in gingipain biogenesis in *P. gingivalis* [27]. Because sialidase is involved in the sialylation of gingipains, a sialidase deficiency in *P. gingivalis* may influence the production, maturation, or secretion of gingipain. We also found that the gingipain activity of both strains was affected by pH, extreme temperatures and oxidative stresses, suggesting that the activities of gingipains require a suitable pH value and a moderate temperature and are weakened under oxidative stress.

LPS is the major constituent of the outer membrane of *P. gingivalis* and is considered to be a major virulence factor of *P. gingivalis*. LPS plays an important structural role and mediates interactions with the microenvironment [28]. Its biological activity is very complex, but the most important activity is to stimulate target cells to secrete large amounts of inflammatory cytokines, leading to tissue destruction. LPS consists of lipid A, a distal polysaccharide (or O-antigen) and a non-repeating “core” oligosaccharide [29]. Lipid A, which is embedded in the outer membrane [28], is the biologically active region of LPS that can cause deregulation of the innate immune system through regulation of the toll-like receptor 2 and 4 pathways [30]. The sialylation of lipooligosaccharides occurs in microorganisms, such as *Haemophilus influenzae* [31], and sialidase may participate in *P. gingivalis* LPS biosynthesis or the sialylation modification. In our study, we compared the LPS activities of the *P. gingivalis* W83 and ∆PG0352 strains grown under different stressful conditions and observed no significant differences between the two strains under any assayed condition but acidic one. In the chromogenic end-point TAL assay, *Tachypleus* amebocyte lysate is primarily agglutinated with lipid A, which is the primary active site of LPS. We speculated that a sialidase deficiency in *P. gingivalis* may influence LPS biosynthesis or the sialylation modification, but that this may not involve lipid A. Stressful conditions, including alkaline conditions, extreme temperatures and oxidative stress did not influence LPS activity. It is noteworthy that the LPS activity of the *P. gingivalis* W83 strain in acidic conditions was increased compared to those in other culture conditions and was also higher than in the ∆PG0352. We speculate that *P. gingivalis* can self-protect against an adverse environment by increasing the activity of LPS to attack the host cells in acidic conditions and that this activity is associated with sialidase. Thus, the activity of LPS in the sialidase-deficient mutant strain cannot increase in acidic conditions, and this mechanism is being further studied in our laboratory.

During biofilm formation, cells must adapt to environmental changes and be able to maintain an appropriate balance between planktonic and biofilm phases. Single-species biofilm formation requires intercellular signaling and cells have a gene transcription profile that is distinct from that of planktonic cells [32]. For *P. gingivalis*, the level of sialidase gene expression is much higher in biofilms than that in planktonic cells [33], suggesting that sialidase is associated with *P. gingivalis* biofilm formation. In this study, live/dead bacterial viability fluorescent staining technology and CLSM were carried out to investigate the ability of *P. gingivalis* to form a biofilm under different conditions and compared the biofilm formation between the *P. gingivalis* W83 and ∆PG0352 strains. We found that the ∆PG0352 formed less biofilm (in terms of thickness and density) in both normal (in agreement with our previous findings [7]) and also stressful culture conditions. There are three possible reasons for this observation. First, sialic acid is an important component of biofilm [34] and the sialidase-deficient *P. gingivalis* strain could not obtain enough sialic acid to form an intact biofilm. Second, the sialidase deficiency in *P. gingivalis* decreased the activity of gingipains, which have been reported to regulate its biofilm formation [35]. Lastly, sialylation of components on the cell surface can help biofilm formation. A previous study showed that sialylation of lipooligosaccharides promotes biofilm formation by nontypeable *Haemophilus influenzae* [31]; Bloch et al. found that the role of surface glycosylation in the closely related oral pathogen *Tannerlla forsythia*, as a factor affecting structural aggregation of *P. gingivalis* in multispecies biofilms [36]. The sialidase-deficient strain could not sialylate the macromolecules on its surface, and its biofilm formation ability was less than that of the wild-type strain. We hypothesized that biofilm formation of *P. gingivalis* would increase to protect cells under stress conditions, but the results of this study showed that compared to *P. gingivalis* under normal culture conditions, both *P. gingivalis* the W83 and ∆PG0352 strains formed less biofilm under stress conditions. There are two possible explanations for this observation. The first is that both strains showed lower growth under these conditions, and the second is that these stressful conditions regulate the expression of *P. gingivalis* proteins, which associated with biofilm formation. For example, the gingipains and hemagglutinins of *P. gingivalis* may affect both the amount of monospecies biofilm formation and the composition of multispecies oral biofilms formation [35, 37, 38]. In addition, the biofilms of both strains were dispersed under oxidative stress, primarily because high concentrations of hydrogen peroxide can permeate into the biofilms and destroy them.

## Conclusion

In the present study, we observed that the survival, virulence and biofilm formation of a sialidase-deficient mutant of *P. gingivalis* is decreased under stressful environmental conditions compared to those of the W83 strain. We speculate that the inhibition of *P. gingivalis* sialidase using a sialidase inhibitor will reduce the survival, virulence and biofilm formation in *P. gingivalis*, which would be a novel therapy for periodontal disease.

## Abbreviations

CLSM: Confocal laser scanning microscope
Kgp: Lys-gingipain
LPS: Lipopolysaccharide
PBS: Phosphate-buffered saline
Rgp: Arg-gingipain
TEM: Transmission electron microscope
TSB: Trypticase soy broth
ΔPG0352: Sialidase-deficient *porphyromonas gingivalis* strain
